# Evaluation of systemic involvement of Coronavirus disease 2019 through spleen; size and texture analysis

**DOI:** 10.3906/sag-2009-270

**Published:** 2021-06-28

**Authors:** Abdussamet BATUR, Abidin KILINÇER, Fatih ATEŞ, Nazlım Aktuğ DEMİR, Recai ERGÜN

**Affiliations:** 1 Department of Radiology, School of Medicine, Selcuk University, Konya Turkey; 2 Department of Infectious Disease and Clinical Microbiology, School of Medicine, Selcuk University, Konya Turkey; 3 Department of Chest Diseases, School of Medicine, Selcuk University, Konya Turkey

**Keywords:** COVID-19, spleen size, texture analysis

## Abstract

**Background/aim:**

To investigate the changes in the spleen size, parenchymal heterogeneity, and computed tomography (CT) texture analysis features of patients diagnosed with Coronavirus disease 2019 (COVID-19)

**Materials and methods:**

The size and parenchymal structure of the spleen in 91 patients who underwent thoracic CT examination due to COVID-19 were evaluated. For the evaluation of parenchymal heterogeneity, CT texture analysis was performed using dedicated software (Olea Medical, France). The texture analysis of each case consisted of 15 first-order intensity-based features, 17 gray level co-occurrence matrix-based features, and 9 gray level run length matrix-based features.

**Results:**

A total of 91 patients (45 males, 46 females) with a mean age of 54.31 ± 16.33 years (range: 18–81) were included in the study. A statistically significant decrease in spleen size was seen in the follow-up CT examinations (p < 0.001) whereas no statistically significant difference was found between the Hounsfield unit (HU) values. The radiomics consisted of first-order intensity-based features such as 90th percentile, maximum, interquartile range, range, mean absolute deviation, standard deviation, and variance, all of which showed statistically significant differences (p *-* values: < 0.001, < 0.001, 0.001, 0.003, 0.001, 0.001, and 0.004, respectively). “Correlation” as a gray level co-occurrence matrix-based feature and “gray level nonuniformity” as a gray level run length matrix-based feature showed statistically differences (p-values: 0.033 and < 0.001, respectively).

**Conclusions:**

Although COVID-19 manifests with lung involvement in the early stage, it can also cause systemic involvement, and the spleen may be one of its target organs. A decrease in the spleen size and parenchymal microstructure changes can be observed in the short follow-up time. It is hoped that the changes in the parenchymal microstructure will be demonstrated by a noninvasive method: texture analysis.

## 1. Introduction

Coronavirus disease 2019 (COVID-19) has spread fast from China and can now be found everywhere in the world. Most of the patients have mainly displayed pneumonia-associated symptoms such as fever, cough, dyspnoea, and myalgia [1]. However, the disease may also involve other organs, such as the gastrointestinal, nervous, and cardiovascular systems [1,2]. It uses angiotensin-converting enzyme 2 (ACE2) as a host cell receptor [3]. Li et al. [1] reported that the lungs had a moderate expression of ACE2 among all the tissues in their study whereas the spleen had the lowest ACE2 expression levels. The spleen is the largest peripheral lymphoid organ in the human body. It is the main site for immune responses and immune cell placement, with blood filtering, blood storage, hematopoietic, and immunomodulation functions [2]. 

Computed tomography (CT) texture analysis is a relatively new image post-processing technique that uses mathematical methods to analyze the distribution and arrangement of all the pixels in the medical images, and has a series of quantitative features. First-order characteristics such as intensity, heterogeneity, deviation, and skewness from the pixel intensity histogram (expressed in Hounsfield units, HU) and second-order characteristics such as the gray level co-occurrence matrix (GLCM) and the gray level run length matrix (GLRLM) parameters describe the spatial relationship and characteristics of the tissue voxels in greater detail [4]. They are commonly used for the noninvasive characterization and grading of tumors. Texture analysis struggles to characterize complex visual patterns by quantitatively identifying simpler but characteristic subpatterns [5–7]. Image texture gives us information about the spatial arrangement of intensities in an image or selected region of an image. To analyze an image texture in computer graphics, there are two ways to approach the issue: structured approach and statistical approach. A structured approach sees an image texture as a set of primitive pixels in some regular or repeated pattern. A statistical approach sees an image texture as a quantitative measure of the arrangement of intensities in a region [8]. The analysis provides information regarding the periodicity of signal intensity patterns generated in images of tissues. Alterations in these signal patterns appear to correlate with histopathological changes in the region of interest [9].

In this study, we investigated the changes in the spleen size, HU density, and CT texture analysis features of patients diagnosed with COVID-19.

## 2. Materials and methods

### 2.1. Patients

This retrospective study was approved by local ethics committee and written informed consent was waived. Between 21 March 2020 and 10 May 2020, 91 consecutive patients with diagnosis of COVID -19, that was made by positive test for COVID-19 viral RNA in nasopharyngeal or oropharyngeal-swab specimens collected from patients by real-time reverse transcription polymerase chain reaction, was enrolled. COVID-19 positive patients with at least two consecutive thoracic CT images, showing imaging findings compatible with COVID-19 [10], were included in the study. Exclusion criteria were; patients with malignancy, chronic disease or hematological disease, patients receiving hormone or immunosuppressive therapy for any reasons, and severe motion artifact on chest CT.

### 2.2. CT technique

All patients underwent chest CT for lung parenchyma evaluation, as a part of our hospital COVID-19 guidelines. All chest CT acquisitions were performed with the patients in supine position and with breath-holding following inspiration, without contrast medium injection. After obtaining a frontal scout view, lung scanning was performed from the apex to the diaphragm using the multislice computed tomography device with 16 detectors [Somatom Emotion 16-slice; Siemens AG Berlin and Munchen-Germany], dedicated only to patients with COVID-19. The following technical parameters were used: tube voltage: 110–120 kV; tube current modulation 100–250 mAs; slice thickness: 3 mm; spiral pitch factor: 0.8. Images were obtained with both mediastinal [width 350 HU; level 50 HU] and parenchymal [width 1400 HU; level –600 HU] window settings. Because only more than half of the spleen could be obtained from the images, the longest axis of the spleen was taken as reference when calculating its size. Since the imaging protocols were predetermined for COVID-19 patients, the amount of spleen for the same patient could be obtained similarly in all CT images. 

### 2.3. Image analysis

A total of 91 patients were included in this study. The thoracic CT examinations in our picture archiving and communication system (PACS) archive were evaluated. Two radiologists with approximately 10 years’ experience each (AB, AK) reviewed the images independently, with the final finding reached by consensus when there was a discrepancy. In the early stages of the epidemic, serial CT scans were performed in some patients, possibly due to the changing clinical situation. We evaluated those who had more than 4 (n = 50) thoracic CT examinations to determine the change in the size of the spleen within 1 week of the initial examination, within 2 weeks of it, and 2 weeks after it. For those who had fewer than 4 examinations (n = 41), the size of the spleen and the time interval between the initial and the last thoracic CT examinations were noted. If the time interval between the 2 CT examinations was 1 week or less (31 of 91 patients), 2 weeks or less (36 of 91 patients), or more than 2 weeks (24 of 91 patients), the specified time intervals were classified into groups 1, 2, and 3 *,* respectively. 

In the axial CT examination, the longest axis of the spleen was measured in the mediastinal window. Then, at the slice level, where the spleen length was measured, HU measurements were made with round ROIs, with the same mm2 in the initial and last CT examinations. We measured the HU values, which we evaluated to investigate the structural change in the parenchyma, only in the first and last CT examinations. We did this to observe the ultimate change in the splenic parenchyma. The ROI areas differed among the patients but were equally determined in the initial and last CT examinations of the same patient. The spleen diameters and HU values were compared to determine if there was a significant difference between them.

The degree of radiological involvement of the lung parenchyma was also evaluated. Presence of ground-glass opacities, presence of consolidation, presence of nodules, and increased reticular interstitial thickness or peribronchovascular thickness were considered parenchymal involvement features. Each of the five lung lobes per patient was assessed on the basis of the following scale according to the distribution of the affected parenchyma: 0 = normal; 1 = *<* 25% abnormality; 2 = 25%–50% abnormality; 3 = 50%–75% abnormality; and 4 = *>* 75% abnormality. An overall lung “total severity score” was reached by summing up the five lung lobe scores (range of possible scores: 0–20) [11], and the difference between the values in the initial and last CTs of the same patient was evaluated.

**Figure 1:**
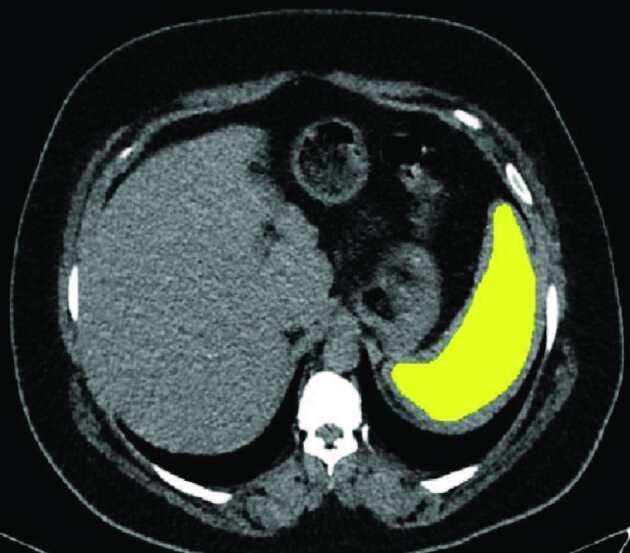
Axial nonenhanced CT showing the region of interest [ROI] was drawn manually to cover the entire spleen parenchyma. Care was taken not to extend the ROI limits a few millimeters from the periphery of the spleen parenchyma to avoid possible errors.

After that, the DICOM data were transferred to a dedicated workstation (Olea sphere v. 3 SP2, Olea Medical, La Ciotat, France). Nonenhanced axial CT images were used to calculate the texture analysis features. At the slice level where the spleen length was measured, an ROI was manually drawn to cover the entire spleen parenchyma on the axial CT images (Figure 1). Care was taken not to extend the ROI limits a few millimeters from the periphery of the spleen parenchyma to avoid possible errors. The texture analysis of each ROI consisted of 15 first-order intensity-based features (entropy, minimum, 10th percentile, 90th percentile, maximum, mean, median, interquartile range, range, mean absolute deviation, standard deviation, skewness, kurtosis, variance, and uniformity), 17 gray level co-occurrence matrix-based features (autocorrelation, joint average, cluster prominence, contrast, correlation, difference average, difference entropy, joint energy, joint entropy, inverse difference moment, inverse difference, inverse variance, maximum probability, sum average, and sum entropy), and 9 gray level run length matrix-based features (short-run emphasis, long-run emphasis, gray level nonuniformity, run percentage, gray level variance, run variance, run entropy, low gray level run emphasis, high gray level run emphasis). The texture analysis features obtained in the 2 CT examinations were compared to determine if there was a significant difference between them.

### 2.4. Statistical analysis

The Kolmogorov–Smirnov test was used to determine the normal distribution of continuous variables. Categorical variables were expressed as raw numbers and quantitative variables were expressed as median [min-max] for nonnormalized variables and as mean ± SD for normal distribution. Comparisons between groups were performed using repeated-measures ANOVA, one way ANOVA, Mann–Whitney’s U and Kruskal–Wallis tests for quantitative variables, as appropriate. Dunn’s pairwise comparison test was used after Kruskal–Wallis test and Tukey test was used after multiple measures ANOVA for further multiple comparisons with Bonferroni correction. P-values inferior to 0.05 was accepted denoting statistical significance. Statistical analysis was performed with SPSS v. 23.

## 3. Results

A total of 91 patients (45 males, 46 females) with a mean age of 54.31 ± 16.33 years (range: 18–81) were included in the study. According to the grouping made on the basis of the time interval between the two CT examinations, there was no statistically significant difference between the patients’ age and sex for all the 3 groups. 

**Figure 2:**
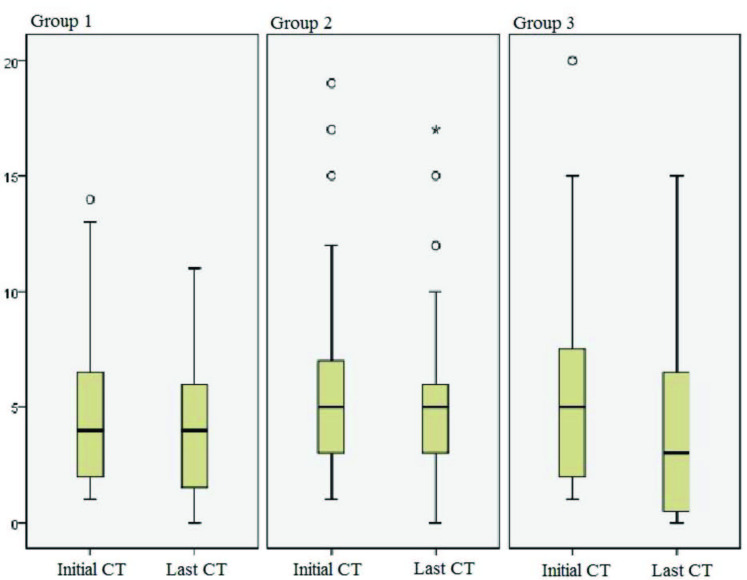
Box plot comparing the groups made according to time interval between the initial and last CT examinations. The heterogenity between groups supports that the distribution was made randomly.

The lung parenchymal involvement was graded as stated in the methods section. The groups’ grades according to the time interval between the CT examinations were compared. There was heterogenity between the groups, which shows that there was random distribution (Figure 2). In the comparison between the first and last CT examinations, lung involvement was found to be progressive in 14 patients, regressive in 55 patients, and stable in 22 patients. However, there was no statistically significant difference between the three groups in the groupings by time interval (Table 1).

**Table 1:** Distribution of spleen size and density and lung involvement scores between groups.

		Group 1 [n = 31]	Group 2 [n = 36]	Group 3 [n = 24]	p
Spleen diameter[mm][mean ± SD]	Initial CT	113.2 ± 18.8	115.5 ± 19.1	110.2 ± 19.2	0.571
	Last CT	108 ± 19.2	105.2 ± 19.2	98.9 ± 17.7	0.208
Diameter difference [mm] [median[min/max]]		5[–7/14]	9.5[–7/45]	12[–9/22]	0.001*
Spleen density [Hounsfield unit][mean ± SD]	Initial CT	47.1 ± 5.6	46.8 ± 5.9	48.8 ± 5.3	0.378
	Last CT	47.9 ± 5.1	49.4 ± 5.2	49.1 ± 5.5	0.499
Lung involvement degree [0–20] [median[min/max]]	Initial CT	4[1/14]	5[1/19]	5[1/20]	0.641
	Last CT	4[0/11]	5[0/17]	3[0/15]	0.195

*Diameter difference = Spleen diameter [initial CT] – spleen diameter [last CT].

**Figure 3:**
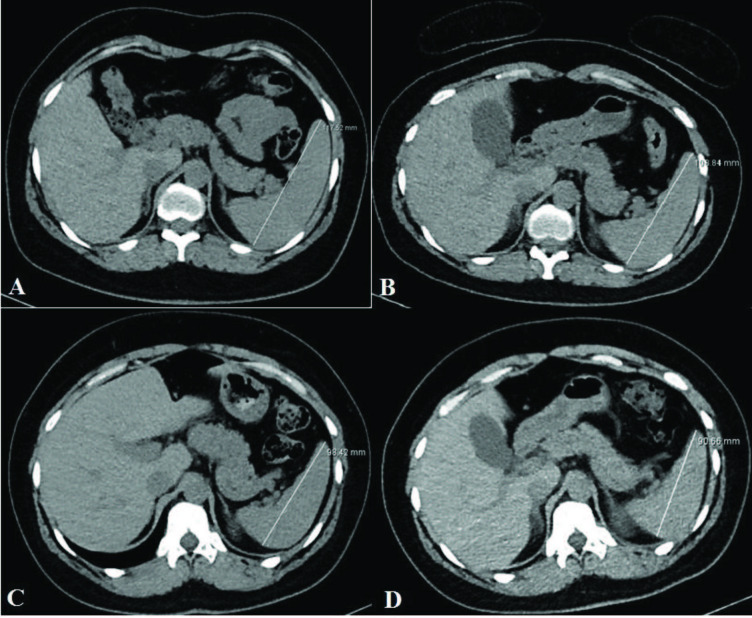
Axial nonenhanced CT showing the longest diameter of the spleen at admission [a], within 1 week [b], within 2 weeks [c], and 2 weeks after [d] the initial CT, respectively.

**Figure 4:**
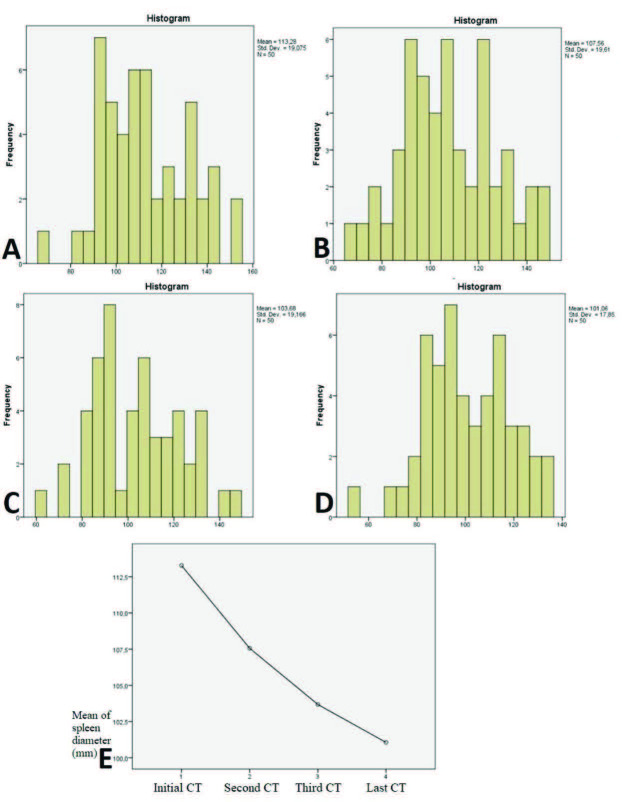
Histogram analysis showing the change in spleen size over time. The longest diameter of the spleen at admission [a], within 1 week [b], within 2 weeks [c], and 2 weeks after [d] the initial CT, respectively. [e] Profile plot showing the change in size-time curve.

The changes in the spleen parenchymal density and spleen diameters between the two CT examinations were evaluated. There was heterogeneity in and between the groupings by time interval, which shows that there was random distribution. The average density values and diameters of the three groups between the two CT examinations were not different (Table 1). There was no statistically significant difference between the HU values. However, there was a statistically significant decrease in the spleen sizes (p < 0.001). The longest diameters of the spleen at admission, within 1 week, within 2 weeks, and 2 weeks after the initial CT were 113.28 ± 19.07 mm, 107.56 ± 19.61 mm, 103.68 ± 19.16 mm, and 101.06 ± 17.85 mm, respectively (Figure 3a-d). There was a statistically significant decrease in spleen size over time (Figure 4a-e). 

**Table 2:** First- and second-order texture analysis parameter values of spleen parenchyma in initial and last CT examinations.

First order	Initial CT	Last CT	p
Entropi	5.02[4.02/5.65]	5.00[4.47/5.57]	0.762
Minimum	–18[–154/22]	–27[–123/12]	0.064
10th percentile	27[–6/40]	24[–56/46]	0.212
90th percentile	66[55/108]	70[59/99]	0.001*
Maximum	108[70/218]	121[79/199]	0.001*
Mean	46.47[30.80/73.18]	48.02[14.79/66.13]	0.080
Median	46[30/73]	48[2/66]	0.168
Interquartile range	22[11/48]	25[13/101]	0.001*
Range	139[44/372]	149[68/292]	0.003*
Mean absolute deviation	13.43[4.62/29.12]	15.05[7.40/51.14]	0.001*
Standard deviation	16.91[7.34/36.94]	19.15[9.30/59.12]	0.001*
Skewness	0.001[–0.975/0.183]	-0.013[–0.371/0.287]	0.457
Kurtosis	3.17[2.8/9.5]	3.18[1.99/4.46]	0.092
Variance	286.08[53.81/1364.19]	366.89[86.5/524.044]	0.004*
Uniformity	0.037[0.029/0.074]	0.037[0.024/0.054]	0.517
Gray level co-occurrence			
Autocorrelation	1090.2[695.8/2165.9]	1127.5[706.0/1797.8]	0.962
Joint average	32.7[26.2/46.5]	33.2[26.4/42.3]	0.938
Cluster prominence	89869.5[17946.9/299918.1]	87344.4[25378.6/761017.8]	0.431
Cluster tendency	166.5[44.9/327.5]	165.3[80.4/634.8]	0.325
Contrast	90.2[20.9/152.1]	95.2[35.5/889.8]	0.153
Correlation	0.311[0.078/0.655]	0.279[0.072/0.894]	0.033*
Difference average	7.5[3.6/9.8]	7.6[4.7/9.5]	0.271
Difference entropy	4.3[3.3/40678]	4.3[3.7/9.7]	0.611
Difference variance	33.6[5.2/56.2]	35.1[13.2/53.7]	0.109
Joint energy	0.002[0.001/0.006]	0.002[0.001/0.003]	0.545
Joint entropy	9.7[7.8/10.1]	9.7[4.0/10.1]	0.774
Inverse difference moment	0.128[0.097/0.243]	0.124[0.100/0.190]	0.353
Inverse difference	0.216[0.179/0.336]	0.212[0.183/0.284]	0.317
Inverse variance	0.129[0.101/0.903]	0.128[0.102/0.199]	0.717
Maximum probability	0.005[0.003/0.017]	0.005[0.001/0.009]	0.362
Sum average	65.4[52.4/92.9]	66.5[52.8/84.6]	0.918
Sum entropy	5.7[6.7/6.2]	5.7[5.2/6.5]	0.225
Gray level run length matrix			
Short run emphasis	0.966[0.472/0.978]	0.967[0.450/0.968]	0.092
Long run emphasis	1.1[0.3/1.3]	1.1[1.1/1.2]	0.175
Gray level nonuniformity	153.3[72.0/417.0]	131.1[34.5/386.5]	0.001*
Run persentage	0.955[0.911/0.970]	0.956[0.451/0.968]	0.133
Gray level variance	69.0[17.4/101.1]	65.6[30.7/168.7]	0.825
Run variance	0.051[0.032/0.107]	0.047[0.035/0.078]	0.051
Run entropy	5.3[4.5/5.6]	5.3[4.8/5.9]	0.852
Low gray level run emphasis	0.002[0.001/0.004]	0.002[0.001/0.004]	0.723
High gray level run emphasis	1138.4[752.7/2176.6]	1178.3[765.7/1819.7]	0.940

The radiomics (texture analysis) values of the initial and last CT examinations obtained for the spleen parenchyma are shown in Table 2. In the comparison between the two examinations, the radiomics consisting of first-order intensity-based features such as 90th percentile, maximum, interquartile range, range, mean absolute deviation, standard deviation, and variance showed statistically significant differences (p-values: < 0.001, < 0.001, 0.001, 0.003, 0.001, 0.001, and 0.004, respectively). “Correlation” as a gray level co-occurrence matrix-based feature and “gray level nonuniformity” as a gray level run length matrix-based feature showed statistically significant differences between the initial and last CT exminations (p-values: 0.033 and < 0.001, respectively). There were no statistically significant differences in the other texture features in the comparison of the initial and last CT examination values.

## 4. Discussion

In this study, we observed that the spleen was also affected in COVID-19 patients other than lung parenchymal involvement. In addition to the reduction in spleen size, we observed differences in texture analysis showing microscopic changes, although there was no difference in macroscopic observation. 

In COVID-19 patients, it is claimed that the virus may enter the peripheral blood from the lungs and cause viremia. Then it attacks the targeting organs that express ACE2 such as spleen, testis, heart, colon, and liver [1,12]. After the virus infects resident, infiltrating, and circulating immune cells, the circulating immune cells carry the virus to other organs. Once COVID-19 enters the spleen, a series of immune responses are initiated and multiple cytokines are released. Both virus and autoimmunity damages the immune cells of the spleen, peripheral and central lymph nodes, and other lymphoid tissues [2,13,14]. In an autopsy study conducted in patients who died due to COVID-19, it was reported that the volume of the spleen decreased significantly [2]. Similar autopsy findings were reported in severe acute respiratory syndrome [SARS] patients, too. Gu et al. reported virus-infected immune cells in the circulating blood, spleen, lymph nodes, and lymphoid tissue of various organs in the autopsy samples. They reported the presence of virus in most of the circulating lymphocytes and lymphoid organs, and atrophy of spleen and lymph nodes in patients with SARS [14].

To our knowledge, this is the first study that evaluates spleen size and parenchyma structure with CT in COVID-19 patients. In our study, it has been observed that the spleen size of patients is reduced consistent with the autopsy reports. In our evaluation of whether the amount of lung parenchymal involvement affects the change in the size of the spleen, we could not find a significant relationship between the two parameters. We observed a decrease in spleen size over time, regardless of changes in the lung parenchyma. The size reduction in the first 2 weeks [p < 0.001] was more significant than after 2 weeks [p = 0.003], although there was no statistically difference. We think that the change in the size of the spleen can be fully observed during long-term follow-up. 

Although there was a significant reduction in the size of the spleen, no significant difference was observed in HU values which shows parenchymal heterogeneity macroscopically. Whereas, it seems to us that we were able to demonstrate the microscopic change in spleen parenchyma through radiomics [texture analysis]. Texture analysis, a computer-assisted detection system, aims to represent pixel distribution, intensities, and dependences using mathematically defined parameters and, thus, can quantitate tissue properties by featuring the characteristics of the medical image [15]. It is generally used at the level of research to demonstrate tumor heterogeneity [16]. Tissue heterogeneity might be associated with cellularity, proliferation, hypoxia, angiogenesis, and necrosis [17]. As mentioned above, both virus and autoimmunity damages the immune cells of the spleen in COVID-19 patients. It seems to us that the changes in texture analysis parameters were due to cellular change and/or splenic parenchymal damage. We think this may reflect microscopic changes in splenic involvement in COVID-19 patients. 

Our study has some limitations. Firstly, we do not have spleen biopsy and hence histopathological data related to involvement. For this reason, we could not reveal the reasons that they may cause shrinkage in size and change in texture analysis. Secondly, since the examinations were directed to the lung, the spleen was not entirely within the field of view. Therefore, volumetric measurement of the spleen could not be made. Thirdly, all cases were COVID-19 positive in radiological imaging and laboratory tests at the time of initial admission. Comparison with the pre-disease period could not be made since CT tests were not available before involvement. Fourthly, because only the lobar involvement amount was taken into account in the scoring and each lobe was evaluated in quarter percentiles, no significant changes were observed although there was a change in the parenchymal involvement parameters and amount. Therefore, lung parenchymal involvement score and hence comparison with the change in the spleen was not satisfactory. Fifthly, since imaging was not performed to asymptomatic and clinically mild cases, possible splenic effect could not be assessed in these patients. Finally, lack of interobserver and intraobserver variability constitutes the limitations of the study.

In conclusion, although COVID-19 manifests itself with lung involvement in early period, it can also cause systemic involvement and the spleen may be one of the target organs. Decrease in the spleen size and parenchymal microstructure changes can be observed in a short follow-up time. It is hopeful that the changes in the parenchymal microstructure may be demonstrated by a noninvasive method, such as texture analysis. Long-term changes in the spleen, shown to be affected in a short time by COVID-19 and other possible systemic involvements, should be kept under observation. 

## Research involving human participants and/or animals

Ethical approval was waived by the local Ethics Committee of Selcuk University in view of the retrospective nature of the study and all the procedures being performed were part of the routine care.

## Informed consent

Informed consent was obtained from all individual participants included in the study.

## Informed consent

This retrospective study was approved by local ethics committee at Selcuk University School of Medicine with the decision numbered 2020/302 and written informed consent was waived.
